# Asc1, Hel2, and Slh1 couple translation arrest to nascent chain degradation

**DOI:** 10.1261/rna.060897.117

**Published:** 2017-05

**Authors:** Cole S. Sitron, Joseph H. Park, Onn Brandman

**Affiliations:** Department of Biochemistry, Stanford University, Stanford, California 94305, USA

**Keywords:** protein quality control, ribosome stalling, translation, CAT tails, Asc1, Hel2, Slh1, Ltn1, Rqc2, Ykr023w, Cue3

## Abstract

Premature arrest of protein synthesis within the open reading frame elicits a protective response that degrades the incomplete nascent chain. In this response, arrested 80S ribosomes are split into their large and small subunits, allowing assembly of the ribosome quality control complex (RQC), which targets nascent chains for degradation. How the cell recognizes arrested nascent chains among the vast pool of actively translating polypeptides is poorly understood. We systematically examined translation arrest and modification of nascent chains in *Saccharomyces cerevisiae* to characterize the steps that couple arrest to RQC targeting. We focused our analysis on two poorly understood 80S ribosome-binding proteins previously implicated in the response to failed translation, Asc1 and Hel2, as well as a new component of the pathway, Slh1, that we identified here. We found that premature arrest at ribosome stalling sequences still occurred robustly in the absence of Asc1, Hel2, and Slh1. However, these three factors were required for the RQC to modify the nascent chain. We propose that Asc1, Hel2, and Slh1 target arresting ribosomes and that this targeting event is a precondition for the RQC to engage the incomplete nascent chain and facilitate its degradation.

## INTRODUCTION

Cells are equipped with multiple mechanisms to identify and respond to defective protein. Aberrant species such as misfolded proteins can be identified via direct interactions with quality-control components such as chaperones and ubiquitin ligases (Hartl and Hayer-Hartl 2009; Shao and Hegde 2016). These sensors recognize unique features of defective proteins (e.g., exposure of hydrophobic patches) and repair or mark them for degradation (Hartl and Hayer-Hartl 2009; Shao and Hegde 2016). Cells are also able to degrade a class of defective protein with no apparent defects: nascent chains arising from stalled mRNA translation that does not terminate normally (Brandman and Hegde 2016). Although these incomplete proteins may contain no intrinsic defects such as misfolding, the cell efficiently identifies them among the much larger actively translating pool and degrades them (Ito-Harashima et al. 2007). Cells must therefore identify defective rounds of translation and take action before the nascent chain leaves the ribosome (Brandman and Hegde 2016). Degradation of the products of stalled translation is conserved throughout Eukarya (Passos et al. 2009; Shao et al. 2013, 2015; Shao and Hegde 2014; Ikeuchi et al. 2016), and failures in this response have been linked to dysregulation of protein homeostasis (Choe et al. 2016; Defenouillere et al. 2016; Yang et al. 2016; Yonashiro et al. 2016) and neurodegenerative phenotypes (Chu et al. 2009; Ishimura et al. 2014).

Stalls in translation occur on the fully assembled 80S ribosome (composed of the 60S and 40S subunits), yet ubiquitylation of stalled nascent chains has been observed on 60S ribosomal subunits that have split from the 40S subunit and mRNA (Brandman et al. 2012; Defenouillère et al. 2013; Shao et al. 2013; Verma et al. 2013). Ubiquitylation is carried out by the conserved ribosome quality control complex (RQC), a set of proteins that binds dissociated 60S-nascent chain complexes and facilitates proteasomal degradation of the incomplete nascent chain and recycling of the 60S subunit (Brandman et al. 2012; Defenouillère et al. 2013). Recent studies have outlined the pathway that leads from the stalled ribosome to RQC-mediated degradation of the nascent chain. First, obstacles such as difficult-to-decode arginine or lysine codons slow translation (Dimitrova et al. 2009; Letzring et al. 2010). From here, translation arrests and the arrest step are irreversibly finalized when ribosome splitting separates the ribosomal subunits from the mRNA (Kobayashi et al. 2010; Shoemaker et al. 2010; Pisareva et al. 2011; Shoemaker and Green 2011; Tsuboi et al. 2012; Shao et al. 2013). RQC engagement with the split 60S-nascent chain complex begins when Rqc2 binds (Defenouillère et al. 2013; Shao et al. 2015). A hallmark of this stage of RQC engagement is modification of the nascent chain by Rqc2, which directs the template-free addition of C-terminal alanine and threonine (CAT tails) to arrested nascent chains (Shen et al. 2015). Binding by Rqc2 stabilizes the association of the E3 ubiquitin ligase Ltn1 (Defenouillère et al. 2013; Shao et al. 2015), which then ubiquitylates the CATylated nascent chain (Bengtson and Joazeiro 2010; Brandman et al. 2012; Shao et al. 2013). The remaining members of the RQC (Rqc1, Cdc48, and its cofactors) may function in extracting the nascent chain from the ribosome for proteasomal degradation (Brandman et al. 2012; Defenouillère et al. 2013; Verma et al. 2013).

Two 80S ribosome-binding proteins, the 40S ribosomal protein Asc1 and E3 ubiquitin ligase Hel2, have been implicated in the initial response to arrested translation that leads to formation of the RQC (Brandman et al. 2012). Asc1 and Hel2 were proposed to be required for translation arrest on synthetic reporter proteins designed to induce stalled translation (Kuroha et al. 2010; Letzring et al. 2013). Such stalling reporters feature a tract of specific codons that induce arrest (Letzring et al. 2010) (e.g., CGA codons encoding arginine) sandwiched between easily translatable mRNA sequences. Translation of stalling reporters prematurely arrests at the stalling sequence, resulting in low levels of the full-length protein product (Dimitrova et al. 2009). The arrested pre-stall protein fragment is then degraded by the RQC and the ubiquitin–proteasome system (Bengtson and Joazeiro 2010; Brandman et al. 2012). While depleting RQC members increased levels of the arrested pre-stall fragment by interfering with its proteasomal degradation (Brandman et al. 2012; Shen et al. 2015), cells lacking Asc1 and Hel2 accumulated more of the full-length reporter product (a fusion of the pre- and post-stall domains) than wild-type (wt) cells (Kuroha et al. 2010; Brandman et al. 2012; Letzring et al. 2013). An “arrest-defect” model has been proposed to explain this result (Kuroha et al. 2010; Inada 2013; Letzring et al. 2013). The arrest-defect model posits that, in the absence of Asc1 and Hel2, impaired translation arrest increases translational read-through past the stall to produce more full-length reporter, which is not subject to proteasomal degradation (Kuroha et al. 2010; Inada 2013; Letzring et al. 2013). Thus, these 80S-binding factors were proposed to induce translation arrest, leading to the ribosome splitting into 60S and 40S subunits. However, it is unclear whether the observed increase in post-stall protein levels is entirely explained by the arrest-defect model or whether Asc1 and Hel2 play another role in the response to failed translation.

We sought to test the arrest-defect model by investigating the steps that link stalled translation to ubiquitylation of the nascent chain in *Saccharomyces cerevisiae*. Our investigation focused on probing the involvement of Asc1 and Hel2 as well as the novel factor Slh1 (implicated in the RQC pathway for the first time here) in these steps. We monitored two events pertinent to degradation of the stalled nascent chain (Brandman et al. 2012; Defenouillère et al. 2013; Shao et al. 2013; Verma et al. 2013): (i) translation arrest to prevent the potentially defective nascent chain from being fully synthesized, and (ii) modification of the nascent chain by the RQC. We found that translation arrests robustly in the absence of Asc1, Hel2, and Slh1. However, these proteins are required for CATylation of the nascent chain. Taken together, our analyses suggest that the arrest-defect model cannot account for the role that Asc1, Hel2, and Slh1 play in facilitating degradation of nascent chains. Instead, we propose that Asc1, Hel2, and Slh1 enable a novel targeting step that permits the RQC to engage the nascent chain.

## RESULTS

### Slh1 is a novel component of the RQC pathway that acts upstream of the RQC

**FIGURE 1.**
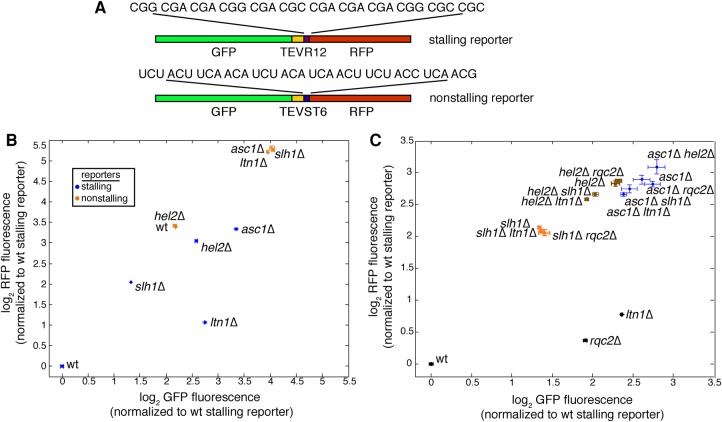
Slh1 is a novel factor in the RQC pathway that acts upstream of the RQC. (*A*) Schematic diagram of the stalling (*above*) and nonstalling (*below*) reporters used in this study. The stalling reporter sandwiches a polyarginine stall sequence between a GFP with a TEV linker and an RFP. The sequence of the nonstalling reporter matches that of the stalling reporter except for the replacement of the polybasic stretch with a serine–threonine linker. Flow cytometry measurements of indicated strains are shown in panels *B* and *C*. (*B*) Mean absolute GFP and RFP fluorescence levels are shown for strains expressing the stalling reporter (blue) and the nonstalling reporter (orange). (*C*) Mean fluorescence measurements of the stalling reporter show the effects of deletion of *ASC1*, *HEL2*, and *SLH1* on *ltn1*Δ and *rqc2*Δ stalling reporter phenotypes. Data are presented as mean ± SEM and are derived from three biological replicates.

The RNA helicase Slh1 was a hit in a previous screen (Brandman et al. 2012) that measured expression of a pre-stall protein fragment of a synthetic stalling reporter; deleting *SLH1* increased levels of the pre-stall protein fragment (Brandman et al. 2012). As is the case for Asc1 and Hel2, Slh1 associates with 80S ribosomes (Daugeron et al. 2011) and its depletion disrupted degradation of the stalling reporter; we wondered whether *slh1*Δ strains have the same stalling reporter phenotype as *asc1*Δ and *hel2*Δ. We therefore analyzed the expression of a similar stalling reporter (Shen et al. 2015) in which a polyarginine stalling sequence is sandwiched between a C-terminal RFP sequence and an N-terminal GFP sequence plus a TEV linker (Fig. 1A). Translation of this reporter arrests at the stalling sequence (Dimitrova et al. 2009), limiting production of a GFP- and RFP-fluorescent full-length protein and instead mostly yielding a truncated, GFP-fluorescent arrest product (Brandman et al. 2012; Shen et al. 2015). This stalled nascent chain is efficiently degraded by the proteasome in a ubiquitin- (Dimitrova et al. 2009) and RQC-dependent (Brandman et al. 2012) manner. To determine whether deleting *SLH1* yields a phenotype similar to deleting an RQC member (more GFP-fluorescent pre-stall protein fragment) or instead yields a phenotype matching deletion of *ASC1* and *HEL2* (more GFP- and RFP-fluorescent full-length protein), we measured the effect of Slh1 depletion on stalling reporter fluorescence. Ablation of *SLH1* increased both GFP and RFP fluorescence relative to wt (Fig. 1B), as did perturbation of *ASC1* or *HEL2* (Fig. 1B). This increase in the amount of full-length reporter protein is the same phenotype that led to the arrest-defect model (Kuroha et al. 2010; Inada 2013; Letzring et al. 2013).

To analyze the roles that Slh1, Asc1, and Hel2 play in responding to failed translation, we sought to rigorously test whether these three factors conform to the arrest-defect model (Kuroha et al. 2010; Inada 2013). The arrest-defect model makes two predictions: (i) in *asc1*Δ, *hel2*Δ, and *slh1*Δ strains, impairing the RQC would have no additional effect on the stalling reporter because the RQC cannot act if arrest does not occur, and (ii) the frequency with which translation continues past the stall sequence of the stalling reporter should rise in *asc1*Δ, *hel2*Δ, and *slh1*Δ strains compared to wt.

To test the first prediction of the arrest-defect model, we compared the levels of the pre-stall protein (GFP) and post-stall protein (RFP) for the stalling reporter in double mutants of *ASC1*, *HEL2*, and *SLH1* with RQC members *LTN1* and *RQC2*. Deleting *LTN1* or *RQC2* had almost no effect in strains lacking *ASC1*, *HEL2*, and *SLH1* (Fig. 1C), in accordance with the arrest-defect model's prediction that Asc1, Hel2, and Slh1 act upstream of the RQC. *ASC1* deletion was dominant to all other perturbations, the effect of *HEL2* deletion was dominant to all perturbations except for *ASC1* deletion, and *SLH1* deletion was dominant only to deletions of *RQC2* and *LTN1* (Fig. 1C). Taken together, these results support the first prediction of the arrest-defect model and suggest that Asc1, Hel2, and the novel factor Slh1 act upstream of the RQC.

### Translation arrest induced by a stalling reporter does not require Asc1, Hel2, or Slh1

**FIGURE 2.**
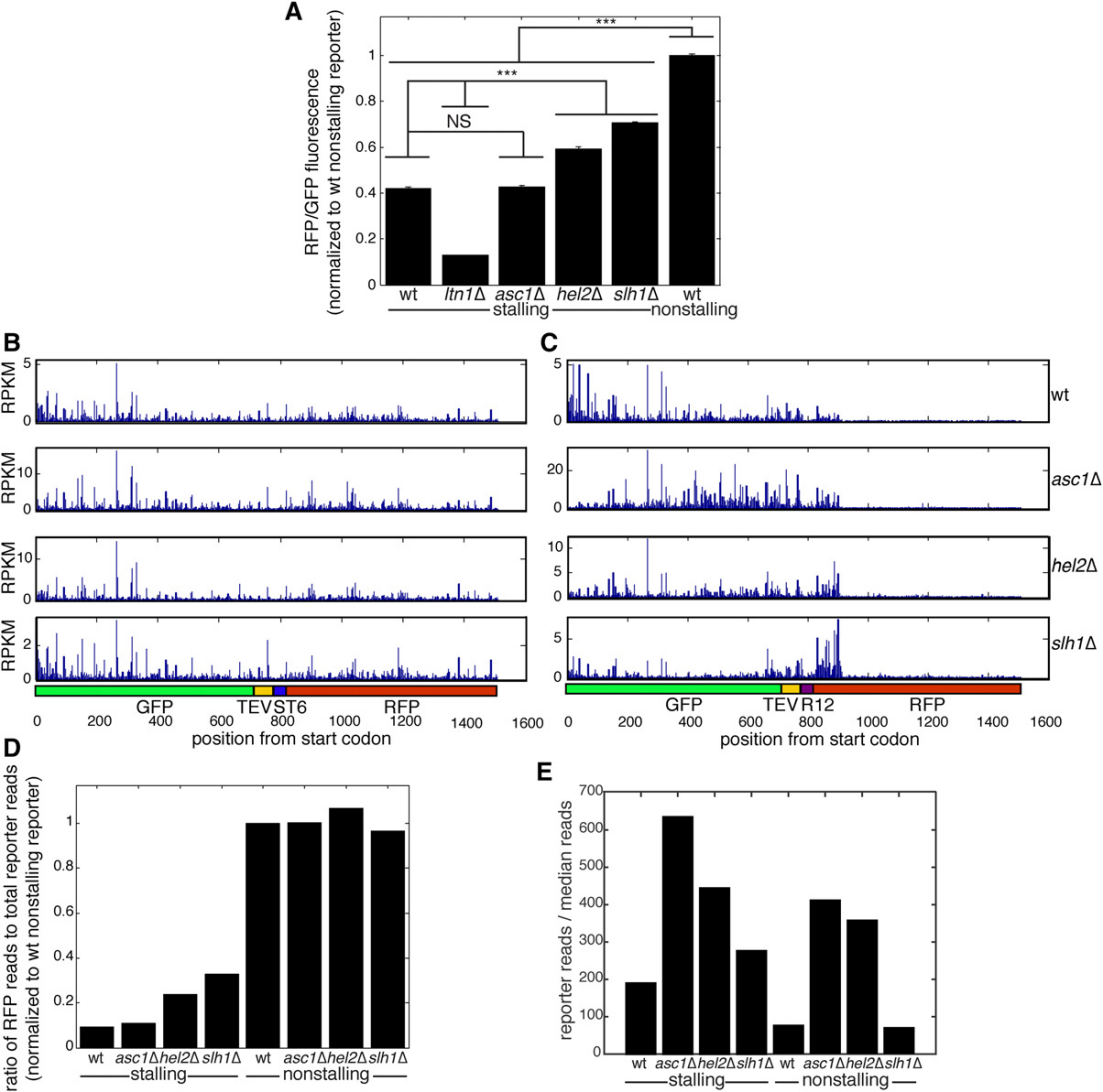
Asc1, Hel2, and Slh1 are not required for translation arrest on a synthetic stalling reporter. (*A*) RFP/GFP fluorescence ratios of stalling and nonstalling reporters in indicated strain backgrounds approximate levels of arrest in *asc1*Δ, *hel2*Δ, and *slh1*Δ strains. For clarity, only statistical comparison between wt and deletion mutant stalling reporter-expressing strains as well as between stalling reporter and wt nonstalling reporter-expressing strains are shown. Data are presented as mean ± SEM. (*n* = 3, one-way ANOVA, [***] *p* < 0.0005, NS = *p* > 0.05.) (*B*,*C*) Ribosome profiling traces are shown for stalling (*B*) or nonstalling (*C*) reporters expressed in indicated strains. Units are reads per kilobase of transcript per million mapped reads (RPKM). (*D*) The relative post-stall occupancy was calculated by dividing ribosome profiling reads per base in the RFP region over total reads per base for the stalling and nonstalling reporters in indicated strains. The first and last 20 codons were excluded to focus analysis on actively translating ribosomes. The region in the 40 codons past the stall was excluded from the actively translating RFP reads (see text). (*E*) mRNA abundance of stalling or nonstalling reporters in the indicated strains was determined by RNA-seq and calculated by normalizing the number of reads over the reporter coding region to the median number of reads for all coding sequences in the transcriptome.

Next, we tested the second prediction of the arrest-defect model: The frequency with which translation continues past the stall sequence of the stalling reporter (“read-through”) should be higher in *asc1*Δ, *hel2*Δ, and *slh1*Δ strains than in wt. We approximated read-through by measuring the ratio of fluorescence from the post-stall protein product (RFP) to the total protein pool (GFP, since both the arrested pre-stall GFP fragment and the GFP-RFP full-length protein contain GFP). The RFP/GFP ratio for the stalling reporter was 42% of a matched control that contained no stalling sequence (Fig. 2A), demonstrating that the RFP/GFP ratio is indicative of read-through. Strikingly, compared to a nonstalling reporter, the RFP/GFP ratio for the stalling reporter was significantly lower in *asc1*Δ, *hel2*Δ, and *slh1*Δ strains (Fig. 2A), suggesting that translation of the stalling reporter did not occur to completion in these genotypes.

Although measuring the steady-state levels of pre-stall and post-stall protein fragments gives some indication of the amount of translation arrest caused by stalling sequences, this measurement's accuracy is limited due to possible differences in the stability and fluorescence of the protein fragments. To circumvent this limitation, we measured ribosome occupancy before and after the reporter's stall sequence. Ribosome profiling (Ingolia et al. 2012) revealed a sharp decrease in the density of translating ribosomes after the stall in wt cells (Fig. 2B,C, top panels). This decrease began 40 codons after the stalling sequence (Fig. 2B,C, top panels). The position of this decrease is consistent with observations that ribosomes slowly translate in the presence of cycloheximide (Hussmann et al. 2015; Requião et al. 2016), which we added before lysis as part of the ribosome profiling protocol. A density of ribosomes at a stall sequence is therefore predicted to be observed immediately downstream from the stall sequence using ribosome profiling. Since the post-stall ribosome density we observe is a result of cycloheximide treatment, we did not assign this density to the population of ribosomes actively translating past the stall. Using this definition, ribosome density was 91% lower past the stall sequence in the stalling reporter compared to the matched nonstalling control (Fig. 2B–D), consistent with the polyarginine sequence robustly arresting translation.

We next tested how deletion of *ASC1*, *HEL2,* and *SLH1* affected read-through of the stalling and nonstalling reporters. Ablation of *ASC1*, *HEL2*, and *SLH1* did not affect the density of translating ribosomes past the nonstalling linker sequence on the nonstalling reporter (Fig. 2B). Consistent with our RFP/GFP fluorescence measurements of the stalling reporter (Fig. 2A), the density of ribosomes past the stall dropped after ablation of *ASC1*, *HEL2*, and *SLH1* (Fig. 2D). The drop in ribosome density past the stall sequence was only weakly diminished in mutants lacking Hel2 and Slh1; the drop in ribosome occupancy past the stall was 76% without Hel2 and 67% without Slh1 relative to 91% in the wt strain and 89% in the *asc1*Δ strain (Fig. 2D). For convenience, we will refer to Hel2 and Slh1 as “weak arrest modulators.” Because we detected a robust drop in ribosome density on the stalling reporter in the absence of Asc1, Hel2, and Slh1, our ribosome profiling measurements do not support the second prediction of the arrest-defect model.

We wondered why deletion of *ASC1* and *HEL2* yielded strong increases in post-stall protein levels in our study (Fig. 1B) and others (Kuroha et al. 2010; Brandman et al. 2012) despite weak effects on translation arrest. Increased mRNA levels could explain these protein-level increases, as higher levels of reporter mRNA could increase absolute post-stall protein production without necessitating any change in arrest. Consistent with this hypothesis, mRNA levels of the stalling reporter increased in *asc1*Δ and *hel2*Δ strains relative to wt (Fig. 2E). Much of the regulation of mRNA levels did not depend on the presence of a stall sequence, since ablation of *ASC1* and *HEL2* also increased nonstalling reporter mRNA levels roughly fivefold over wt (Fig. 2E).

### Asc1, Hel2, and Slh1 are not required for translation arrest induced by polybasic-encoding tracts in the transcriptome

**FIGURE 3.**
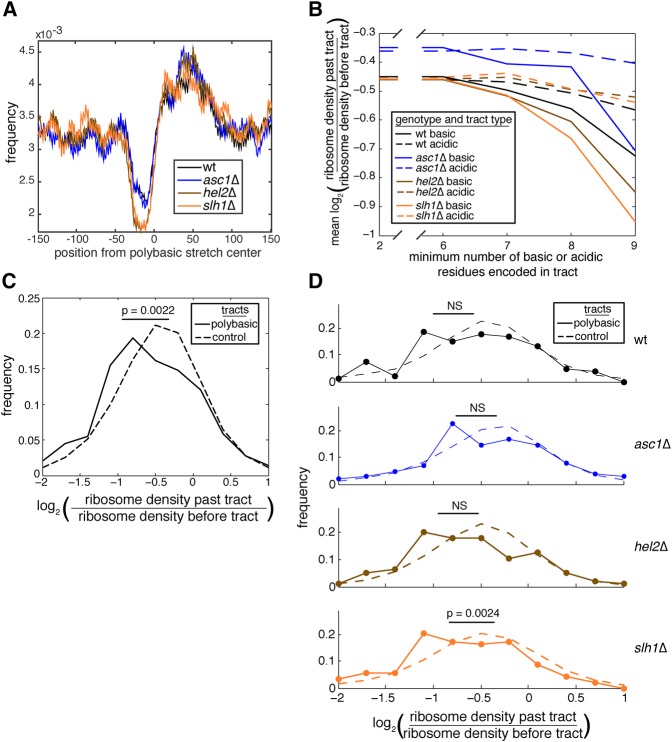
Asc1, Hel2, and Slh1 are not required for translation arrest on endogenous transcripts. (*A*) Ribosome profiling read frequency in the region surrounding polybasic-encoding tracts encoded in the transcriptome is given for all ribosome profiling experiments performed (data pooled from two samples per genotype). Polybasic-encoding tracts were defined as encoding at least six basic residues in a 10-residue window. (*B*) Analysis of ribosome occupancy past tracts encoding acidic or basic residues revealed the effect of increased dosage of encoded residues on drop off in ribosome density in *asc1*Δ, *hel2*Δ, and *slh1*Δ genotypes. Data were pooled from two ribosome profiling samples per genotype. (*C*) Comparison of ribosome occupancy past polybasic-encoding tracts and control tracts on endogenous transcripts in *asc1*Δ, *hel2*Δ, and *slh1*Δ strains as measured by ribosome profiling. Data were pooled from six total samples, two per *asc1*Δ, *hel2*Δ, and *slh1*Δ strains. From these samples, 290 windows from 52 genes were analyzed that encoded eight or greater basic residues in a 10-residue window. Of note, 3,473,215 control windows were used from 4469 genes that encoded two or fewer basic residues in a 10-residue window. Given *p*-value was calculated from a Student's *t*-test. (*D*) Comparison of ribosome density past polybasic-encoding tracts and control tracts on endogenous transcripts in *asc1*Δ, *hel2*Δ, and *slh1*Δ strains as measured by ribosome profiling. Data were pooled from two samples per genotype and come from the same transcripts used in the analysis in *C*. Given *p*-value was calculated as in *C*. (NS = *p* > 0.05.)

To test whether Asc1, Hel2, and Slh1 alter ribosome occupancy at endogenous stalling sequences, we analyzed ribosome density on transcripts that encode polybasic tracts. These polybasic-encoding tracts are predicted to slow translation and therefore may induce translation arrest (Brandman et al. 2012; Charneski and Hurst 2013), similar to the stalling reporter used in this study (Fig. 2D). Other groups measuring the density of ribosomes surrounding polybasic-encoding tracts have observed a drop in density at the polybasic-encoding tract and a concomitant peak after the tract (Hussmann et al. 2015; Requião et al. 2016). Here, we observed the same drop in density at the tract and a peak of density past the tract (Fig. 3A), suggesting that Asc1, Hel2, and Slh1 do not affect the occupancy of ribosomes in the vicinity of polybasic-encoding tracts.

We next sought to measure translation arrest caused by polybasic-encoding tracts. Ribosome density values showed a dose-dependent drop past these stretches when they encoded seven or more basic residues within a 10-residue window (Fig. 3B). Tracts of basic residues induced a stronger drop-off in mean ribosome occupancy past the tract than acidic residues (Fig. 3B), which are not predicted to induce arrest (Dimitrova et al. 2009; Letzring et al. 2010). This drop occurred regardless of the presence of Asc1, Hel2, and Slh1 (Fig. 3B), consistent with translation arrest occurring on endogenous transcripts independently of these factors. We selected transcripts encoding eight or more basic residues in a 10-residue window (“polybasic-encoding tracts”) for further analysis along with those encoding two or fewer as a control (“control tracts”). Control tracts had similar read numbers to polybasic-encoding tracts (Supplemental Fig. S1A). Furthermore, we controlled for additional tract features by sampling control tracts from the same distributions of tract position (Supplemental Fig. S1B) and transcript lengths (Supplemental Fig. S1C) as polybasic-encoding tracts. Polybasic-encoding tracts had a significantly higher drop in mean ribosome occupancy past the tract than did control tracts when drop-off measurements were pooled from *asc1*Δ, *hel2*Δ, and *slh1*Δ strains (Fig. 3C). Within each genotype, we observed that ribosome density dropped more for polybasic-encoding tracts than for control tracts (Fig. 3D). Because sampling was sparser for the nonpooled data set than for the pooled data set, the statistical significance of these drop-off values within genotypes was weaker than that for the pooled measurements but nonetheless followed the same trend (Fig. 3C,D). Therefore, Asc1, Hel2, and Slh1 are likely not required for arrest caused by polybasic-encoding tracts in the *S. cerevisiae* transcriptome. Taken together, our measurements of arrest on the stalling reporter (Fig. 2D) and on endogenous stalling transcripts (Fig. 3A–D) are not consistent with the second prediction of the arrest-defect model and make it unlikely that Asc1, Hel2, or Slh1 are required for translation arrest.

### RQC engagement with stalled nascent chains requires Asc1, Hel2, and Slh1

**FIGURE 4.**
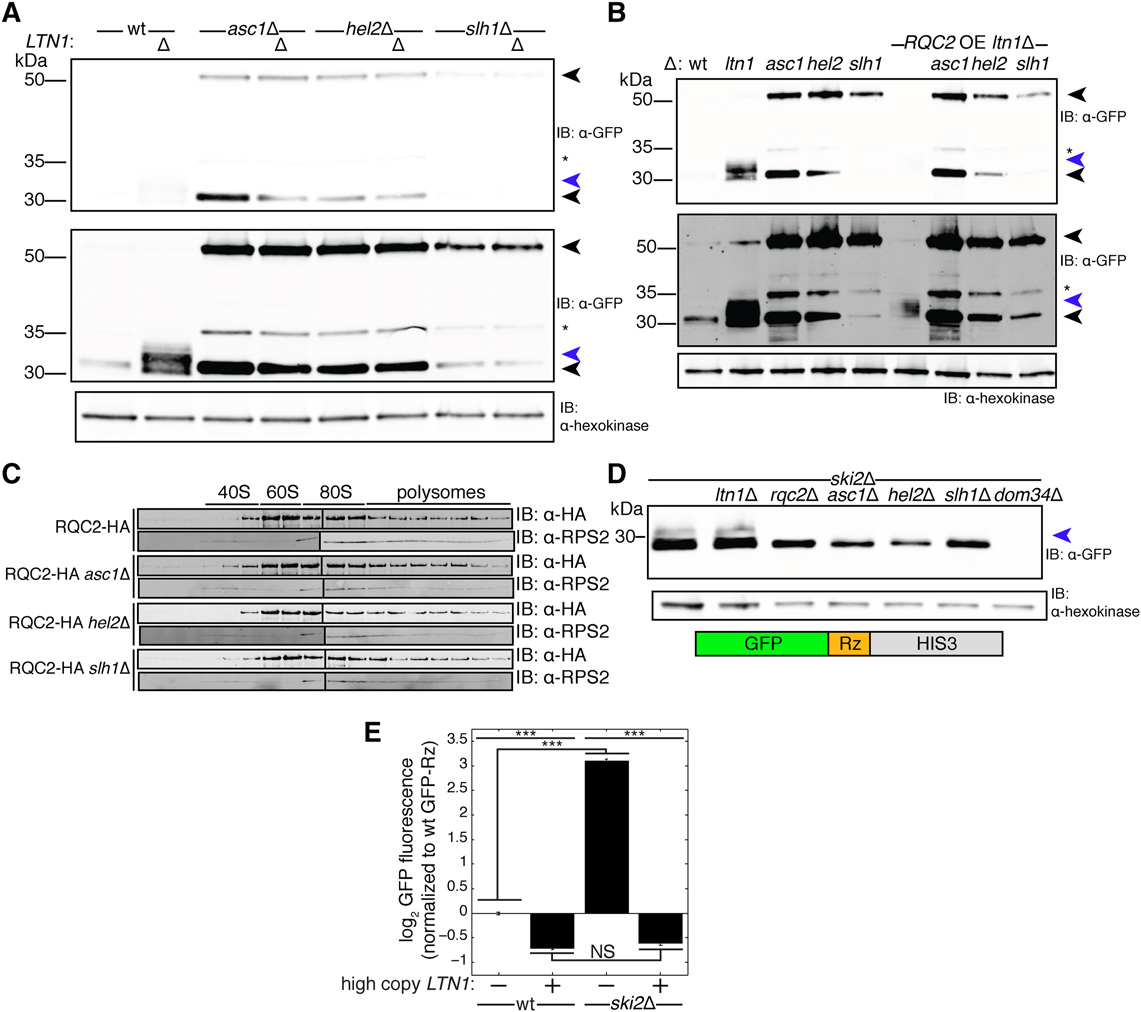
Asc1, Hel2, and Slh1 are required for CATylation of arrested nascent chains. (*A*,*B*,*D*) Whole-cell immunoblots (IB) of the indicated yeast strains expressing the stalling reporter. *Upper* black arrow indicates the full-length translation product and the *lower* black arrow indicates the arrest product. Blue arrow denotes a high-molecular weight smear consistent with CATylation that appears *above* the arrest product. Star denotes a 35 kDa product of the stalling reporter that likely results from proteolytic processing of the full-length translation product RFP in SDS-PAGE (Gross et al. 2000). *Upper* and *lower* anti-GFP panels differ in contrast adjustment. (*A*) Depletion of Asc1, Hel2, and Slh1 tested the requirement of these factors in detection of a higher molecular weight smear *above* the stalling reporter arrest product in *ltn1*Δ strains without (*A*) and with (*B*) *RQC2* overexpression (OE). In panel *B*, the arrest product band in *RQC2* OE *ltn1*Δ appears weak because a large proportion of the reporter is in detergent-insoluble aggregates in this strain (Yonashiro et al. 2016). (*C*) IB of Rqc2-HA and Rps2 (a small ribosomal subunit marker) shows the distribution of Rqc2 in fractions of a 10%–30% sucrose polysome gradient with and without the presence of Asc1, Hel2, and Slh1. (*D*) Whole-cell IB of GFP-Rz reporter (schematic *below*) expressed in the *ski2*Δ background with additional genetic perturbations to assay for a higher molecular weight smear *above* the GFP-Rz arrest product. (*E*) Flow cytometry tested the effects of additional Ltn1 on levels of the GFP-Rz protein product. Data are presented as mean ± SEM. (*n* = 3, one-way ANOVA, [***] *p* < 0.0005, NS = *p* > 0.05.)

Epistasis experiments suggested that Asc1, Hel2, and Slh1 act upstream of the RQC (Fig. 1C). However, these factors did not appear to be required for arrest (Figs. 2C,D, 3B–D). Thus, we sought to test whether Asc1, Hel2, and Slh1 enable the RQC to engage nascent chains. A hallmark of RQC engagement with the nascent chain is the addition of CAT tails by Rqc2 (Shen et al. 2015). To assay CATylation, we used SDS-PAGE to detect shifts in mobility of the stalling reporter after depletion of Ltn1 (Fig. 4A, lanes 1,2). If a factor, for instance Rqc2, is required for CATylation, then its absence in an *ltn1*Δ strain would result in a collapse of the CAT-tailed arrest product smear into a single, discrete band (Shen et al. 2015). Strikingly, deletion of *ASC1*, *HEL2*, or *SLH1* abrogated detection of the smear above the stalling reporter arrest product, which ran instead as a more discrete band (Fig. 4A, lanes 3–8). Because we observed an arrest product that did not change in mobility upon Ltn1 depletion, these data are consistent with a failure of CATylation in the absence of Asc1, Hel2, and Slh1 despite arrest occurring.

There are multiple scenarios under which arrest could occur without CATylation in the absence of Asc1, Hel2, or Slh1. One possibility is that Rqc2 becomes limiting in the absence of Asc1, Hel2, or Slh1. If Rqc2 becomes limiting, then increasing the concentration of Rqc2 should at least partially restore detection of an arrest product smear. However, depletion of Asc1, Hel2, and Slh1 still abrogated detection of the arrest product smear when *RQC2* was overexpressed (Fig. 4B, lanes 6–9). Another explanation for loss of CATylation could be that Rqc2 cannot be recruited to dissociated 60S-nascent chain complexes. We observed that the association of Rqc2 with 60S fractions in a polysome gradient did not depend on Asc1, Hel2, or Slh1 (Fig. 4C). Therefore, the apparent defects in CATylation we observed upon ablation of *ASC1*, *HEL2*, and *SLH1* do not appear to be caused by limiting Rqc2 or a general defect in Rqc2 association with 60S ribosomal subunits.

We wondered whether the possible requirement for Asc1, Hel2, and Slh1 for CATylation depends on a specific mode of arrest or splitting that occurs only in the presence of these factors. Under the confines of such a model, arrest and splitting pathways relying on these factors would produce a 60S-nascent chain that can be engaged by Rqc2. In this model, putative alternative pathways that take over and lead to the arrest observed in *asc1*Δ, *hel2*Δ, and *slh1*Δ strains do not result in a 60S-nascent chain competent for Rqc2 to engage.

To test this model, we analyzed a verified RQC substrate, GFP-Rz, that arrests without the need of *trans*-acting factors and is split by a Dom34-dependent pathway (Tsuboi et al. 2012; Matsuda et al. 2014). GFP-Rz contains a hammerhead ribozyme sequence that causes self-cleavage of the mRNA (Düvel et al. 2002) after the GFP sequence, resulting in a stop codon-less transcript. Ribosomes translating such a transcript necessarily arrest at the end of the message and become substrates of the Dom34-dependent splitting pathway (Kobayashi et al. 2010; Shoemaker et al. 2010; Pisareva et al. 2011; Shoemaker and Green 2011; Tsuboi et al. 2012; Shao et al. 2013). Therefore, translation arrest on GFP-Rz occurs because ribosomes reach the extreme end of the message and a Dom34-dependent pathway splits the resultant arrested ribosome. If Asc1, Hel2, and Slh1 initialize an RQC-specific arrest and splitting mode, then CATylation of GFP-Rz should not be detected.

To analyze the CATylation status of GFP-Rz, we monitored the SDS-PAGE mobility of GFP-Rz in *ski2*Δ strains, which have an impaired exosome that permits expression of truncated mRNAs like GFP-Rz (Meaux and Van Hoof 2006; Tsuboi et al. 2012). Surprisingly, the GFP-Rz arrest product was robustly detected as an Rqc2-dependent smear in the presence of intact Ltn1 (Fig. 4D, lanes 1,3), which normally marks proteins modified by Rqc2 for degradation (Shen et al. 2015). We hypothesized that the protein fragment escaped degradation and was able to be detected because increased levels of endogenous truncated transcripts, like GFP-Rz, increase the burden of substrates on Ltn1, causing Ltn1 to become limiting. Consistent with this hypothesis, expressing additional Ltn1 on a high-copy plasmid eliminated the GFP-Rz protein product to levels below wt (Fig. 4E).

To verify that ribosomes translating GFP-Rz are split by a Dom34-dependent pathway, we deleted *DOM34* in the *ski2*Δ strain and monitored GFP-Rz expression. Consistent with published results (Kobayashi et al. 2010; Tsuboi et al. 2012), expression of GFP-Rz was strongly down-regulated in the absence of Dom34 (Fig. 4D, lane 7). This observation is consistent with Dom34 preventing a “traffic jam” of static ribosomes by splitting ribosomes arrested at the end of this message (Kobayashi et al. 2010; Tsuboi et al. 2012).

Finally, we determined whether the CATylation-consistent mobility of GFP-Rz depends on Asc1, Hel2, and Slh1 by depleting these proteins in the *ski2*Δ background and monitoring migration of GFP-Rz by SDS-PAGE. Strikingly, detection of a higher molecular weight smear above the GFP-Rz arrest product depended on all of these proteins (Fig. 4D, lanes 4–6). Detection of this arrest product in the absence of Asc1, Hel2, and Slh1 suggests that a traffic jam of ribosomes does not occur, and that Dom34 can split ribosomes arrested by GFP-Rz independently of these factors. Therefore, GFP-Rz appears to induce translation arrest and ribosome splitting independently of Asc1, Hel2, and Slh1, yet the mobility of the arrest product suggests that CATylation still occurs and relies on these three factors.

### Ykr023w and Cue3 copurify with Hel2 and Slh1 and their depletion similarly affects arrest

**FIGURE 5.**
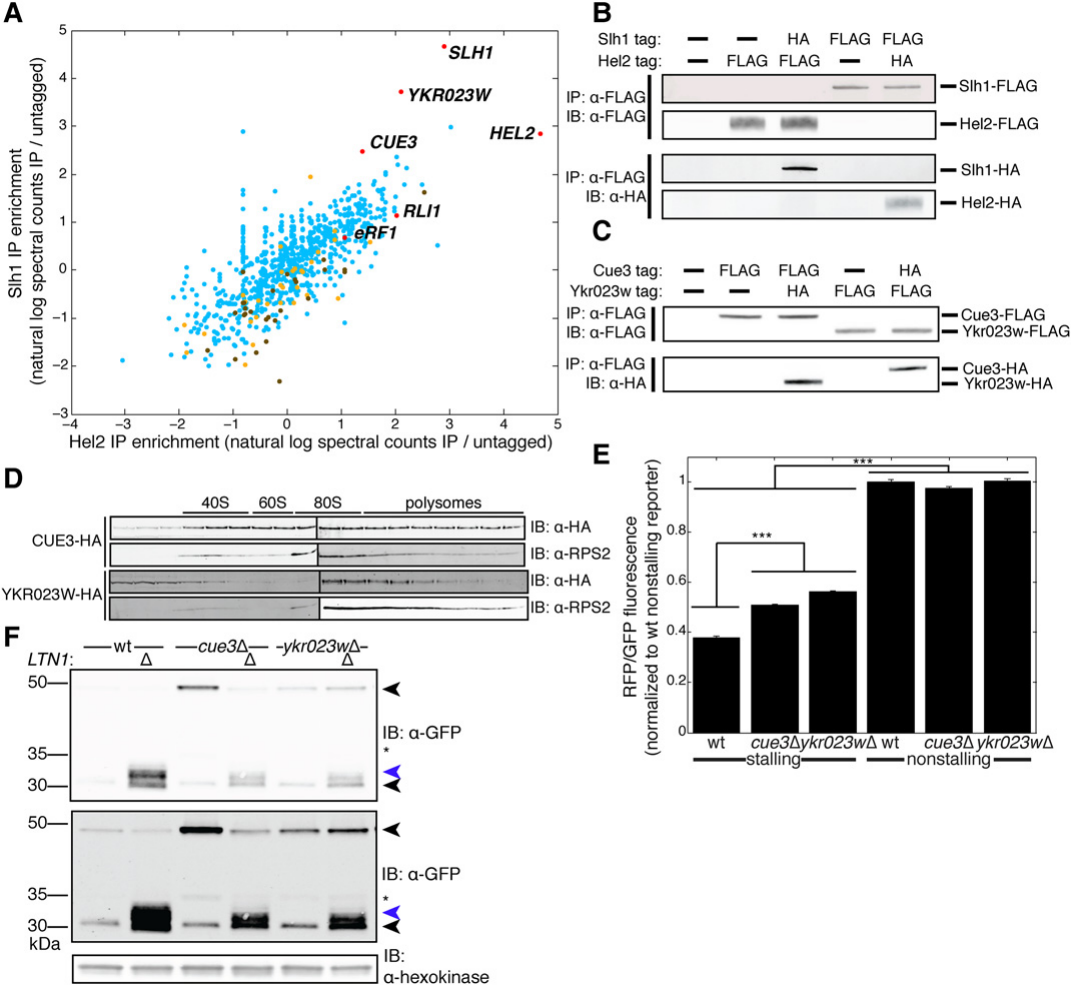
Ykr023w and Cue3 copurify with Hel2 and Slh1 and their depletion similarly affects arrest. (*A*) Enrichment of all proteins detected by mass spectrometry in Hel2 and Slh1 immunoprecipitations (IPs). Proteins of interest are highlighted in red and denoted with their gene names. Large ribosomal subunit proteins are denoted in yellow and small ribosomal subunit proteins in brown. Enrichment is presented as the mean from two biological replicates. For the full data set, see Supplemental Table S1. (*B,C*) Immunoblots (IBs) of IPs of Hel2 and Slh1 (*B*) as well as Cue3 and Ykr023w (*C*) tested the interactions detected by mass spectrometry in *A*. (*D*) IBs of 10%–30% sucrose polysome gradient fractions, probing for Cue3-HA, Ykr023w-HA, and Rps2 (a marker for small ribosomal subunit proteins) showed the distribution of Cue3 and Ykr023w in ribosomal fractions. (*E*) RFP/GFP fluorescence ratios measured by flow cytometry on the indicated yeast strains expressing stalling and nonstalling reporters. For clarity, only statistical comparisons between wt and deletion mutant stalling reporter-expressing strains as well as between stalling reporter and matched-background nonstalling reporter-expressing strains are shown. Data are presented as mean ± SEM. (*n* = 3, one-way ANOVA, [***] *p* < 0.0005, NS = *p* > 0.05.) (*F*) IB of the stalling reporter in *cue3*Δ and *ykr023w*Δ strains tested whether Cue3 and Ykr023w are required for the stalling reporter arrest product to run as a smear upon *LTN1* ablation. *Upper* black arrow indicates the full-length translation product and the *lower* black arrow indicates the arrest product. Blue arrow denotes a high molecular weight smear consistent with CATylation. Star indicates a 35 kDa proteolytic product of the stalling reporter likely resulting from cleavage in SDS-PAGE (Gross et al. 2000). *Upper* and *lower* anti-GFP panels differ in contrast adjustment.

We next investigated whether additional proteins act alongside Asc1, Hel2, and Slh1 to respond to stalled translation. To identify these proteins, we immunoprecipitated versions of these proteins tagged in their endogenous, genomic context and determined their binding partners via mass spectrometry (Fig. 5A, full data set in Supplemental Table S1). We did not immunoprecipitate Asc1, as it is stoichiometrically bound to ribosomes (Inada et al. 2002) and therefore its purification would not be as specific to stalled ribosomes as that of Hel2 and Slh1. This data set revealed that Hel2 and Slh1 coimmunoprecipitated (Fig. 5A), which we confirmed via immunoblotting (Fig. 5B). Additional species enriched in these IPs included 40S and 60S ribosomal subunit proteins as well as the canonical splitting factors eRF1 and Rli1 (see Discussion) (Fig. 5A). Cue3, a ubiquitin-binding protein (Shih et al. 2003), and Ykr023w, an uncharacterized protein whose mammalian ortholog functions in a complex with the Slh1 ortholog (Jung et al. 2002), were highly enriched interactors of Hel2 and Slh1 (Fig. 5A). Cue3 and Ykr023w also physically interacted with each other on co-IP and immunoblot (Fig. 5C). Neither of these factors has previously been implicated in a cotranslational quality control process. Like Hel2 and Slh1, both of these proteins associate with translating ribosomes, as they sedimented along with polysomal fractions in a polysome gradient (Fig. 5D). Similarly to *HEL2* and *SLH1*, ablation of *CUE3* and *YKR023W* weakly increased the RFP/GFP ratio for the stalling reporter (Fig. 5E), suggesting a mild defect in translation arrest. Unlike Hel2 or Slh1, the depletion of Cue3 and Ykr023w did not prevent the arrest product from running as a smear in *ltn1*Δ strains (Fig. 5F). While we did detect the arrest product smear in *cue3*Δ*ltn1*Δ and *ykr023w*Δ*ltn1*Δ genotypes, the smear was less prominent than in the *ltn1*Δ genotype (Fig. 5F). Taken together, these data suggest that the novel factors Cue3 and Ykr023w physically interact with each other and with Hel2 and Slh1; although their depletion weakly alleviates arrest, CATylation can still occur.

## DISCUSSION

Here, we have characterized the functions of a cast of proteins that enable cells to degrade arrested nascent chains. We found that the novel RQC pathway component Slh1, along with Asc1 and Hel2, acts upstream of the RQC (Fig. 1C). Translation arrested in the absence of Asc1, Hel2, and Slh1 (Figs. 2A–D, 3B–D), but these factors were required for the RQC to CATylate the nascent chain (Fig. 4A). CATylation depended on Asc1, Hel2, and Slh1 even on RQC substrates that induce a Dom34-dependent splitting pathway (Fig. 4D). We propose that Asc1, Hel2, and Slh1 couple translation arrest to CATylation by facilitating a novel round of targeting that enables the RQC to engage the nascent chain.

Epistasis experiments suggested that Asc1, Hel2, and Slh1 act upstream of the RQC (Fig. 1C). Our study of the function of these three proteins began by investigating their role in translation arrest, the initial step in the pathway that leads to the RQC. Multiple experimental approaches led us to conclude that polybasic-encoding tracts arrest translation even in the absence of Asc1, Hel2, and Slh1. We first measured production of the pre- and post-stall protein fragments flanking a polyarginine sequence in a fluorescent stalling reporter (Fig. 1A). By flow cytometry, the ratio of the post-stall fragment levels to total reporter protein levels was less than a nonstalling control in each strain we tested (Fig. 2A), indicating that arrest had occurred. Next, we quantified ribosome occupancy past the polybasic-encoding tracts of the stalling reporter and endogenous transcripts. Ribosome occupancy dropped after polybasic-encoding tracts on our stalling reporter (Fig. 2C,D) and on endogenous transcripts in *asc1*Δ, *hel2*Δ, and *slh1*Δ genotypes (Fig. 3B–D). Fluorescence measurements tended to underestimate arrest when compared to ribosome profiling (Fig. 2A compared to Fig. 2D). This discrepancy could be due to quenching of the pre-stall GFP domain by the post-stall RFP domain or due to differences in stability or fluorescence of reporter protein fragments. We directly observed a stalling reporter protein fragment in wt, *asc1*Δ, *hel2*Δ, and *slh1*Δ genotypes by SDS-PAGE (Fig. 4A). The molecular weight of this fragment was consistent with translation abortion at the polyarginine tract (Fig. 4A). Although translation of the stalling reporter arrested less in *hel2*Δ and *slh1*Δ strains than in wt and *asc1*Δ (Fig. 2A,D, 4A), fluorescence, ribosome profiling, and SDS-PAGE measurements each indicated that translation arrested robustly in the absence of Asc1, Hel2, and Slh1.

As was found in previous studies (Kuroha et al. 2010; Brandman et al. 2012), we observed that depletion of Asc1 and Hel2 led to increases in the absolute levels of a protein encoded past a stall by fluorescence (Fig. 1B) and by immunoblot (Fig. 4A). While this phenotype was proposed to be caused by a defect in arrest (Kuroha et al. 2010; Inada 2013; Letzring et al. 2013), Asc1 and Hel2 do not appear to be strong modulators of arrest (Fig. 2A,D). Instead, we found that ablation of *ASC1* and *HEL2* strongly increased mRNA levels for both stalling and nonstalling reporters (Fig. 2E). These mRNA level increases could account for how Asc1 and Hel2 depletion increased absolute levels of protein encoded past stalls (Figs. 1B, 4A; Kuroha et al. 2010; Brandman et al. 2012) without abolishing translation arrest (Figs. 2A–D, 3B,C). By using ratiometric measurements of the stalling reporter (Fig. 2A,D), we were able to quantify arrest and control for misleading effects on absolute reporter mRNA and protein expression levels (Figs. 1B, 2E).

We observed a dramatic drop off in translating ribosomes after stalls in the absence of Asc1, Hel2, or Slh1 (Fig. 2C), suggesting that most stalled ribosomes are being split off the message by a pathway that is independent of these three factors. Although the canonical splitting machinery (eRF1(Sup45)-eRF3(Sup35)-Rli1) normally dissociates ribosomes terminating at a stop codon, eRF1–eRF3–Rli1 can act to dissociate ribosomes when translation is abnormally slow (Chiabudini et al. 2014) and eRF1–eRF3–Rli1 can generate dissociated 60S-nascent chain complexes when ribosomes are stalled internally on an mRNA (Shcherbik et al. 2016). Consistent with the canonical splitting machinery dissociating stalled ribosomes, we found that the weak arrest modulators Hel2 and Slh1 copurified with eRF1 and Rli1 (Fig. 5A). Our inability to detect Dom34 in these purifications does not preclude Dom34 having a role in dissociating arrested ribosomes in the RQC pathway. Indeed, Dom34 may have escaped detection due to its lower abundance in cells than eRF1 and Rli1 (Ghaemmaghami et al. 2003). In addition to splitting ribosomes at the extreme end of transcripts, Dom34 has been shown to dissociate ribosomes stalled at poly-lysine sequences within the poly(A) tail (Guydosh and Green 2014) and within the ORF (Guydosh and Green 2017). Further work will be needed to determine the stall types that each of these splitting modes preferentially responds to in order to generate substrates for the RQC.

CATylation failed without Asc1, Hel2, or Slh1 on substrates that induce stalls internally on an mRNA (Fig. 4A) and substrates that induce stalls due to mRNA truncation (Fig. 4D). The second class of substrates generates a stalled translation complex that is split by a Dom34-dependent pathway to produce a 60S-nascent chain (Kobayashi et al. 2010; Shoemaker et al. 2010; Pisareva et al. 2011; Shoemaker and Green 2011; Shao et al. 2013). The failure of CATylation on the split 60S-nascent chain suggests that the RQC has failed to engage the arrested nascent chain. Thus, targeting of the nascent chain by the RQC might not solely rely on production of a split 60S-nascent chain, as was previously proposed. We propose that RQC engagement with nascent chains relies on an Asc1-, Hel2-, and Slh1-dependent targeting step, which may account for how the absence of Asc1, Hel2, and Slh1 disabled CATylation (Figs. 1C, 4A,D) without eliminating arrest (Figs. 2A–D, 3B–D) or splitting (Fig. 2C). The failure of Hel2 and Slh1 to target arresting translation complexes may result in read-through of the stall in some cases, as we observed a weak defect in arrest in strains lacking these proteins (Fig. 2A,D). While depletion of Cue3 and Ykr023w yielded a similar weak arrest phenotype to Hel2 and Slh1 depletion (Figs. 2A, 5E), Cue3 and Ykr023w were not required for CATylation (Fig. 5F). The weak arrest defect in *cue3*Δ and *ykr023w*Δ genotypes is a likely explanation for lower levels of CATylated arrest product in these strains (Fig. 5F). The existence of an Asc1-, Hel2-, and Slh1-mediated targeting event would not preclude other target selection steps such as accommodation of the splitting machinery (Shao et al. 2013; Shao and Hegde 2016) or recognition of the 60S-nascent chain complex that results from splitting of the arrested ribosome (Shao et al. 2015; Shao and Hegde 2016). Determining how Asc1, Hel2, and Slh1 contribute to nascent chain targeting is an exciting area for future research. Further investigation of this response and its regulatory factors may improve the understanding of how failed translation is linked to disease (Chu et al. 2009; Ishimura et al. 2014).

## MATERIALS AND METHODS

### Yeast strain construction and culture

All deletions and integrations were made by transformation of BY4741 background yeast by standard methods. These transformations used PCR products using primers with 40 bp homology with the genome on the 5′ end and plasmids containing a NATMX6, KANMX6, or HIS3MX6 selection cassette alone (in the case of deletions) or a selection marker along with a coding sequence (in the case of endogenous carboxy-terminal epitope tags). The plasmid stalling reporter (GTRR) was described by Shen et al. (2015), and plasmid GFP-Rz (GFP-Rz-Flag-HIS3) was described by Kobayashi et al. (2010) and was a gift from Dr. T. Inada. The high-copy *LTN1* plasmid (pLTN1-LTN1-tLTN1-URA3-2µ) and the nonstalling reporter (pTDH3-GTSTR-tADH1-URA3) were cloned during the course of this study.

All yeast cultures were grown at 30°C in YPD or synthetic defined media with appropriate nutrient drop-outs.

### Flow cytometry

Flow cytometry was performed in biological triplicate using a BD Accuri C6 Flow Cytometer (BD Biosciences). All presented single-color fluorescence measurements were side-scatter normalized. All raw fluorescence values were normalized to a wt control lacking any reporter prior to further computation. Unless otherwise stated, values appearing in the figures are the mean with error bars representing standard error of the mean for three biological replicates. Log-phase cultures were grown in synthetic defined media containing appropriate nutrient drop-outs.

### Ribosome profiling

Ribosome profiling was performed as described previously (Ingolia et al. 2012). Yeast cultures were treated with 100 µg/mL cycloheximide (Thermo Fisher Scientific) before harvest and RNase I-digested (Thermo Fisher Scientific) monosomes were collected by sucrose density centrifugation. A wide band centered at 28 bp was used as input into library construction. The resultant library was sequenced on a HiSeq 4000 (Illumina). Reads were mapped using bowtie 1.1.1 (Langmead et al. 2009) and the *Saccharomyces* Genome Database (yeastgenome.org).

For the analysis of ribosome occupancy around polybasic-encoding tracts, read frequency was computed in the window plus and minus 150 nt from the center of the polybasic-encoding tract. We analyzed windows encoding at least six lysines or arginines in a 10-residue stretch. To avoid overlapping windows within 50 residues of each other, we selected the window encoding the most lysines or arginines for analysis. The reads in each window were normalized to the sum of reads in the window. Windows with less than 10 reads were excluded.

For the analysis of ribosome occupancy past the polybasic-encoding tracts, control transcripts were defined as encoding two or fewer lysines or arginines in a 10-residue stretch. To eliminate bias caused by the positions of polybasic-encoding tracts, control tracts were sampled from the same distribution of positions along the transcript as the polybasic tract-encoding transcripts. Transcripts that received <25 reads in a wt sample, contained the tract of interest in the first or last 5% of the length of the transcript, or had <30 codons between the end of the polybasic tract and the stop codon were excluded from analysis to minimize noise. To calculate ribosome occupancy past the tract of interest, length-normalized reads past the tract were divided by length-normalized reads from before the tract. To minimize artifacts caused by cycloheximide pretreatment on densities at polybasic-encoding tracts, the region after the tract was defined as occurring from 40 codons past the last basic residue (Hussmann et al. 2015; Requião et al. 2016) to 20 codons before the stop codon (Ingolia et al. 2011); the region before the tract was defined as occurring from 20 codons after the start (Ingolia et al. 2011) to the beginning of the region defined as after the tract. Transcripts that had variations in the ratio of post-stall to pre-stall occupancy greater than two standard deviations past the mean of the control distribution were excluded from analysis to reduce noise.

### RNA-seq

RNA-seq libraries were prepared from acid phenol:chloroform-extracted (Thermo Fisher Scientific) RNA using the Scriptseq v2 Kit (Illumina) and sequenced on a HiSeq 2500 (Illumina). Reads were mapped as for ribosome profiling.

### Immunoprecipitation (IP) and polysome analysis

Log-phase yeast cultures were harvested using vacuum filtration and flash frozen in liquid nitrogen before lysis by cryo-grinding. Extract was prepared from cryo-ground yeast by thawing 1:1 (for IP) or 1:2 (for sucrose gradients) in “IP buffer” adapted from Defenouillère et al. (2013) containing 20 mM HEPES K pH 7.4 (Thermo Fisher Scientific), 100 mM KOAc (Thermo Fisher Scientific), and 10 mM MgCl_2_ (Thermo Fisher Scientific) containing EDTA-Free Pierce Protease Inhibitor Mini Tablets (Thermo Fisher Scientific).

IPs were carried out via a 1-h incubation in Anti-FLAG M2 Affinity Resin (Sigma-Aldrich) with rotation at 4°C. Flag elutions occurred over 1 h with 1 mg/mL 3xFlag peptide (Stanford Protein and Nucleic Acids Facility) in IP buffer with stirring on ice (for mass spectrometry or sucrose gradients). A 10%–30% gradient of sucrose in IP Buffer with 100 µg/mL cycloheximide fractionated clarified lysate through ultracentrifugation for 3 h at 35,000 rpm and 4°C in SW41-Ti-compatible tubes (Seton Scientific).

Fractions from sucrose gradients were collected using a Biocomp Gradient Station (Biocomp Instruments). Absorbance at 254 nm was continuously monitored and recorded using a UA-6 Detector (Teledyne Isco) and Gradient Profiler Software version 2.07 (Biocomp Instruments). Fractions were denatured directly in sample buffer.

### Immunoblots

Yeast cultures were spun down and lysed by boiling in sample buffer. The whole-cell extract or sucrose fractions were run on Novex NuPAGE 1.5 mm 4%–12% bis–tris gels (Thermo Fisher Scientific) with the Spectra BR Ladder (Thermo Fisher Scientific) as a molecular weight reference. Gels were transferred onto 0.45 µm nitrocellulose (Thermo Fisher Scientific), blocked in 5% fat-free milk (Safeway) in TBST, and stained using 1:2000 Pierce mouse anti-GFP (Thermo Fisher Scientific), rabbit anti-Hexokinase (US Biological), rabbit anti-RPS2 (Aviva Systems Biology), Pierce mouse anti-HA (Thermo Fisher Scientific), or Pierce rabbit anti-Flag (Thermo Fisher Scientific), then stained with 1:5000 IRDye 800CW donkey anti-mouse (Li-Cor Biosciences), IRDye 800CW donkey anti-rabbit (Li-Cor Biosciences), IRDye 680RD goat anti-rabbit (Li-Cor Biosciences), or IRDye 680RD goat anti-mouse (Li-Cor Biosciences). Immunoblots were scanned on a Li-Cor Odyssey (Li-Cor Biosciences).

### Mass spectrometry

Eluted IPs were denatured in sample buffer, run on Novex NuPAGE 1.5 mm 4%–12% bis–tris gels, excised, trypsin digested in-gel, and analyzed by the Stanford University Mass Spectrometry Facility using a Thermo Oribtrap Fusion Tribrid (Thermo Fisher Scientific). Proteins were identified using Byonic proteomics search pipeline (Protein Metrics) and the *Saccharomyces* Genome Database (yeastgenome.org). Enrichment for each protein was calculated by: (i) averaging the protein's total peptide spectral counts (+1 to make log-scale data into rational numbers) normalized by total spectral counts between two biological replicates, then (ii) dividing the average spectral count-normalized value by the same value in the nontagged control IP.

### Statistical analysis

For all flow cytometry data, statistical comparisons between deletion mutants and reporter-matched controls were carried out by analysis of variance (ANOVA) followed by Tukey's honestly significant difference test. For ribosome profiling data, differences between distributions of ribosome occupancy measurements were tested for significance by a Student's *t*-test.

## SUPPLEMENTAL MATERIAL

Supplemental material is available for this article.

## Supplementary Material

Supplemental Material
