# Supplementary material for: Asc1, Hel2, and Slh1 couple translation arrest to nascent chain degradation
Source: RNA. 2017 May;23(5):798–810. doi: 10.1261/rna.060897.117 (PMC5393187; doi:10.1261/rna.060897.117)
Supplement: Supplemental Material [file supp_23_5_798__index.html]

Asc1, Hel2, and Slh1 couple translation arrest to nascent chain degradation — Supplemental Material 

# Asc1, Hel2, and Slh1 couple translation arrest to nascent chain degradation

## Supplemental Material

- Supplemental\_FigureS1\_Legend.docx
- Supplemental\_FigureS1.ai
- Supplemental\_TableS1.xlsx
