# Supplementary material for: Asc1, Hel2, and Slh1 couple translation arrest to nascent chain degradation
Source: RNA. 2017 May;23(5):798–810. doi: 10.1261/rna.060897.117 (PMC5393187; doi:10.1261/rna.060897.117)
Supplement: Supplemental Material [file supp_060897.117_Supplemental_FigureS1_Legend.docx]

Figure S1. Arrest occurs due to polybasic-encoding stretches in the absence of Asc1, Hel2, and Slh1. (**A**) Distribution of reads for polybasic-encoding and control transcripts used in ribosome profiling analysis of endogenous transcripts in the pooled *asc1*∆, *hel2*∆, and *slh1*∆ data set in Figure 3C. (**B**) Distribution of tract positions along the length of transcripts used in pooled analysis of *asc1*∆, *hel2*∆, and *slh1*∆ samples in Figure 3C. The distribution from polybasic-encoding transcripts was used to randomly select control tracts at the same relative position along the transcript for analysis. (**C**) Distribution of transcript lengths in *asc1*∆, *hel2*∆, and *slh1*∆ samples used in the analysis in Figure 3C. The length distribution from the polybasic-encoding tracts was used to sample control tracts from transcripts with the same lengths.
