# Supplementary figures and images for: Asc1, Hel2, and Slh1 couple translation arrest to nascent chain degradation
Source: RNA. 2017 May;23(5):798–810. doi: 10.1261/rna.060897.117 (PMC5393187; doi:10.1261/rna.060897.117)

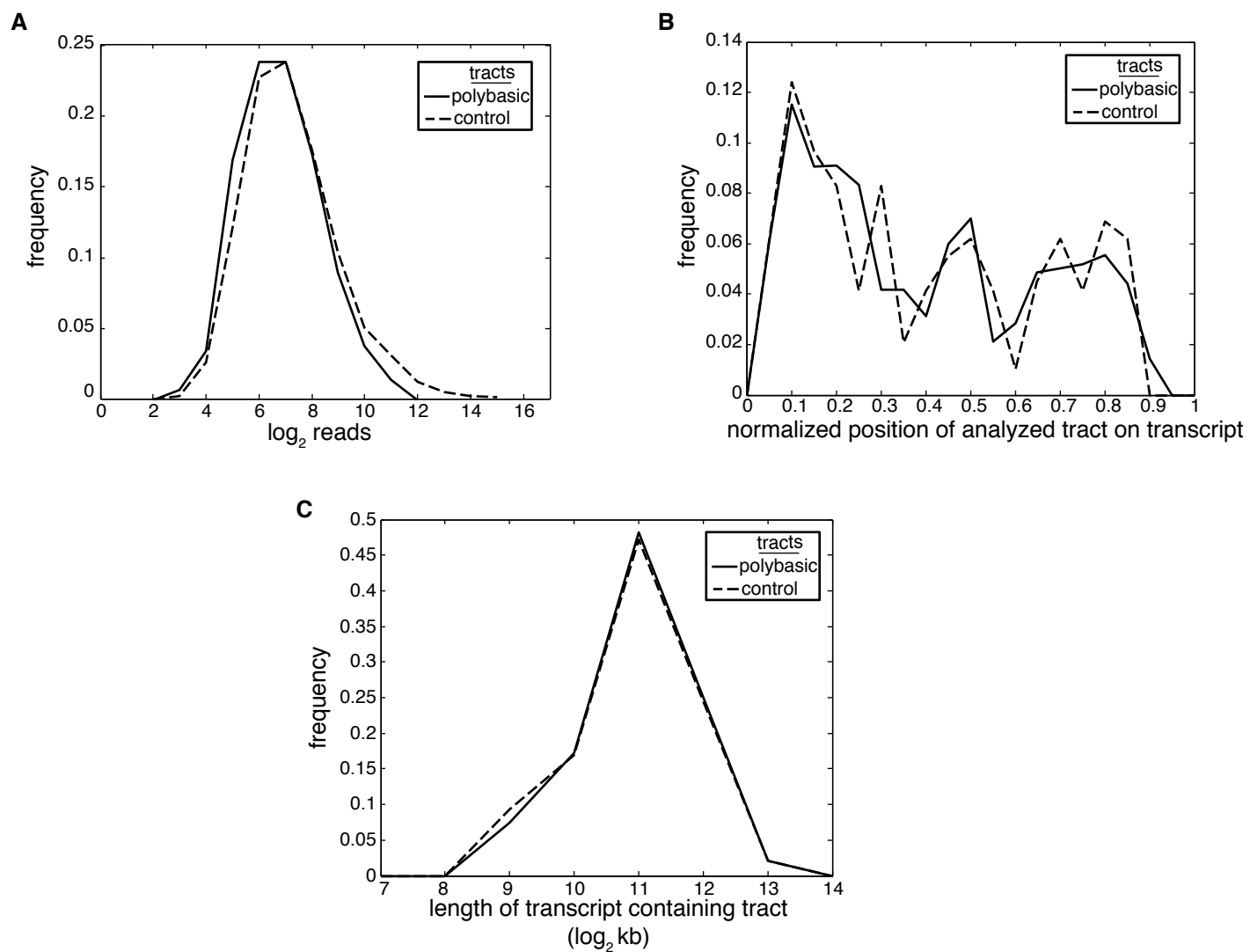

Supplement: Supplemental Material [file supp_060897.117_Supplemental_FigureS1.ps]
